# Assessment of Personal Health Care Management and Chronic Disease Prevalence: Comparative Analysis of Demographic, Socioeconomic, and Health-Related Variables

**DOI:** 10.2196/jmir.8784

**Published:** 2018-10-18

**Authors:** Ryan H Sandefer, Bonnie L Westra, Saif S Khairat, David S Pieczkiewicz, Stuart M Speedie

**Affiliations:** 1 Department of Health Informatics and Information Management College of St. Scholastica Duluth, MN United States; 2 Center for Nursing Informatics School of Nursing & Institute for Health Informatics University of Minnesota Minneapolis, MN United States; 3 Carolina Health Informatics Program University of North Carolina at Chapel Hill Chapel Hill, NC United States; 4 School of Nursing University of North Carolina at Chapel Hill Chapel Hill, NC United States; 5 Institute for Health Informatics University of Minnesota Twin Cities Minneapolis, MN United States

**Keywords:** personal health information, consumer participation, social determinants of health, personal health records

## Abstract

**Background:**

The use of personal health care management (PHM) is increasing rapidly within the United States because of implementation of health technology across the health care continuum and increased regulatory requirements for health care providers and organizations promoting the use of PHM, particularly the use of text messaging (short message service), Web-based scheduling, and Web-based requests for prescription renewals. Limited research has been conducted comparing PHM use across groups based on chronic conditions.

**Objective:**

This study aimed to describe the overall utilization of PHM and compare individual characteristics associated with PHM in groups with no reported chronic conditions, with 1 chronic condition, and with 2 or more such conditions.

**Methods:**

Datasets drawn from the National Health Interview Survey were analyzed using multiple logistic regression to determine the level of PHM use in relation to demographic, socioeconomic, or health-related factors. Data from 47,814 individuals were analyzed using logistic regression.

**Results:**

Approximately 12.19% (5737/47,814) of respondents reported using PHM, but higher rates of use were reported by individuals with higher levels of education and income. The overall rate of PHM remained stable between 2009 and 2014, despite increased focus on the promotion of patient engagement initiatives. Demographic factors predictive of PHM use included people who were younger, non-Hispanic, and who lived in the western region of the United States. There were also differences in PHM use based on socioeconomic factors. Respondents with college-level education were over 2.5 times more likely to use PHM than respondents without college-level education. Health-related factors were also predictive of PHM use. Individuals with health insurance and a usual place for health care were more likely to use PHM than individuals with no health insurance and no usual place for health care. Individuals reporting a single chronic condition or multiple chronic conditions reported slightly higher levels of PHM use than individuals reporting no chronic conditions. Individuals with no chronic conditions who did not experience barriers to accessing health care were more likely to use PHM than individuals with 1 or more chronic conditions.

**Conclusions:**

The findings of this study illustrated the disparities in PHM use based on the number of chronic conditions and that multiple factors influence the use of PHM, including economics and education. These findings provide evidence of the challenge associated with engaging patients using electronic health information as the health care industry continues to evolve.

## Introduction

### Background

Patient access to their electronic health information has been identified as a key priority for improving care quality and efficiency [1,2]. Individual access to and personal use of health information is a cornerstone of recent national health care efforts. As quoted in a study, “With access to their electronic health information, individuals can serve as intermediaries of information exchange among providers and use innovative applications to better manage their health” [3]. The Centers for Medicare and Medicaid Services (CMS) has incorporated electronic access to health information within the electronic health record (EHR) Incentive Program, which requires eligible professionals and hospitals to demonstrate meaningful use of EHR systems. Patient and family engagement is 1 of the 4 primary goals of the EHR Incentive Program, and the program includes multiple measures of patient and family engagement through the use of health information technology. These include sending and receiving secure messages between patients and providers; providing Web-based access to view, download, and transmit health information; and identifying and providing patient-specific educational resources based on clinically relevant information housed within the certified EHR system [1]. The CMS accountable care organization also promotes patient engagement and care coordination in an effort to restrain costs, improve patient experience of care, improve self-management, and facilitate communication between patients and providers [4].

These federal programs require measures of patient and family engagement because of the impact of the individual use of health information on care quality. Multiple studies have identified an association between personal use of health information and improvements in chronic disease management [5-9]. Studies have also shown an association between personal use of health information and improvements in clinical quality outcomes, patient satisfaction, and overall perceptions regarding the efficacy of communication between patients and health care stakeholders [10-15].

In an effort to meet the program goals of the EHR Incentive Program and accountable care organizations, US health care organizations have started implementing EHRs, patient portals, and personal health records at an unprecedented rate. The number of US nonfederal acute care hospitals with the capability of offering patients the ability to electronically view, download, and transmit their health information increased from 10% in 2012 to 69% in 2015. Over 95% of US hospitals provide patients the ability to view their health information electronically [16]. Similarly, between 2013 and 2014, the number of individuals who were offered access to their Web-based medical records increased from 28% to 38%. Over half of the individuals who were offered access to their Web-based medical record in 2014 accessed it [17].

The focus on electronic access to patient health information is not limited to the United States. There are international efforts to provide access to Web-based health information to address issues of access, affordability, and quality. Research on adoption and use of these systems has been conducted in Denmark, Canada, Australia, and Estonia, among others [18-21].

Despite the rapid increase in the capability to view, download, and transmit personal health information, there are disparities regarding individual access and use of their health information. According to one estimate, approximately 4 of 10 US adults used some type of health information technology in 2013, but individuals with less education, lower incomes, or those who lived in rural areas were less likely to email health care providers (HCPs), view laboratory results on the Web, and access health-related information with mobile phones [3]. Similar research has demonstrated disparities in access and use of electronic health information [22-24]. Use of technology for managing personal health information is associated with age [23-27], race [7,24,25,28,29], ethnicity [23-25], and gender [7,23,25]. The socioeconomic factors of income and education level are also related to an individual’s use of technology in accessing and using health information [3,7,24,25,28].

Individuals who report having chronic conditions are more likely to electronically access and use personal health information and are also more likely to access personal health information repeatedly [24,28,30,31]. Nearly half of the US population suffers from at least 1 chronic condition, and nearly 12% of the US population reports having 3 or more chronic conditions [32]. The advantage of using technologies to electronically access health information is more effective management and coordination of care [33]. Individuals managing 1 or more chronic conditions, likely across numerous HCPs and institutions, have the potential to benefit by accessing their information electronically, downloading the information, and sharing it with other members of the care team.

### Objectives

Although individuals with chronic health conditions appear to access electronic health information more frequently than others, there has been little research conducted regarding the relationship between the number of chronic conditions and participation in electronic personal health care management (PHM). In this project, we are interested in how demographic, socioeconomic, and health-related variables affect PHM. PHM, as defined here, refers to the individual use of internet-based technology to access personal health information or communicate with HCPs regarding patient health information. PHM is considered active participation with a health care entity through the use of technology, and PHM refers to use of technology-mediated apps by an individual to assist in meeting her or his health care–related needs. The purpose of this research was to describe the overall utilization of PHM and compare individual characteristics associated with PHM among groups with no reported chronic conditions, with 1 chronic condition, and with 2 or more such conditions.

## Methods

### Sample

Data from the US National Health Interview Survey (NHIS) aggregated by the Integrated Health Interview Series (IHIS) were used for this analysis. The IHIS has collected and harmonized 52 years of NHIS data for the purpose of research and analysis [33]. The Centers for Disease Control and Prevention annually conducts the NHIS “to secure accurate and current statistical information on the amount, distribution, and effects of illness and disability in the United States and the services rendered for or because of such conditions.” The NHIS sample is representative of the US population drawn from each US state and the District of Columbia and includes approximately 35,000 households and 87,500 persons annually. It has an average response rate of approximately 90%, and it has been conducted annually since 1957 [34]. Multiple publications have detailed the use of the NHIS [35,36]. For the purpose of this project, an SAS text file was downloaded from the IHIS portal and imported into the R statistical software package for analysis (R Foundation for Statistical Computing).

NHIS survey data regarding adults older than 18 years from 2009, 2011, 2012, 2013, to 2014 were combined for this study. The 2010 survey was excluded because it did not collect the information regarding PHM required for this analysis. Between 2009 and 2014, there were 605,001 individuals interviewed. The data used in this study were limited to adults who were asked and responded “Yes” or “No” to specific questions regarding PHM and were further limited to only individuals with complete data for the variables included in the analysis, resulting in a study sample size of 50,062 individuals.

### Variable Selection

The NHIS includes items related to a variety of health care–related characteristics, demographics, health conditions, and behaviors. A dichotomous variable labeled PHM was calculated based on the questionnaire items related to text messaging (short message service) HCPs, refilling of prescriptions on the Web, and scheduling of health care appointments on the Web. PHM indicates use of electronic health information management. The levels of responses for each question were “Yes,” “No,” “Refused,” “Not Ascertained,” or “Don’t Know.” A response of “Yes” to any of the 3 questions resulted in a “Yes” PHM response, otherwise a “No” was assigned. For the purpose of the analysis, “No” was coded as 0 and “Yes” was coded as 1. Any response of “Refused,” “Don’t Know,” or “Not Ascertained” to any of the 3 questions resulted in the removal of that respondent from the analysis. Individual use of PHM was the dependent variable. The entire list of predictor variables and their assignable value sets are listed in the Results section.

### Statistical Analysis

The R statistical software package, version 3.2.3, was used for all statistical analyses. The *survey* package allows the analysis to account for complexity of the NHIS sample. To account for combining multiple years of NHIS data, the sample weight was divided by the number of years (5 years) of data included in the analysis [37]. Multiple logistic regression was used to identify predictors of PHM among multiple independent samples based on chronic condition status.

Moreover, 3 different models of PHM were created to characterize and compare the 3 subgroups of respondents. The first model included only respondents who did not report any of the 5 chronic conditions (diabetes, hypertension, asthma, heart condition, or arthritis) and represented the ability to predict PHM for individuals without chronic conditions. The second model included only individuals reported as having 1 of the 5 chronic conditions (one chronic condition). The third model included all observations where respondents reported having 2 or more of the 5 chronic conditions included in this study (multiple chronic conditions).

Descriptive statistics for each group and each predictor variable were also calculated. A 2-sample test for equality of proportions was used to compare PHM use.

### Protection of Human and Animal Subjects

Analysis of the NHIS data was deemed exempt from review by the University of Minnesota Institutional Review Board.

## Results

### Respondent Characteristics

The total NHIS sample for this study after exclusions was 50,061 individuals—the number of total responses without controlling for missing values was 75,305 with 8082 positive PHM responses (10.73%, 8082/75,305). The NHIS sample was separated into 3 mutually exclusive subgroups based on the number of reported chronic conditions. The sizes of the samples were as follows: 22,965 (no chronic condition), 13,325 (1 chronic condition), and 13,771 (multiple chronic conditions).

### Overall Personal Health Care Management Use

**Figure 1:**
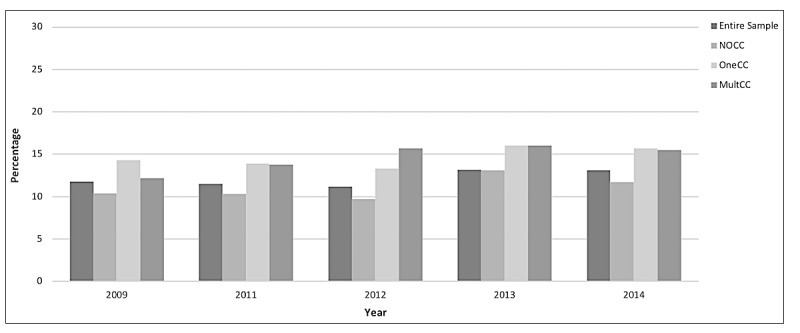
Proportion of US adults using personal health management (PHM) by year by chronic condition group. CC: chronic condition.

The overall proportion of US adults who reported PHM use between 2009 and 2014 was 12.19%, and the use of PHM has increased slightly over that period; Figure 1 provides a breakdown by group and by year. Overall, there was an increase in reported PHM use from 11.24% to 13.27% between 2012 and 2013.

PHM use for each subgroup characterized by demographic, socioeconomic, and health-related characteristics are listed in Tables 1-3.

The overall level of use of PHM varied slightly by group. The highest overall rates of PHM use were among individuals reporting 1 chronic condition. The results show that 14.97% (1996/13,325) individuals with a single chronic condition reported PHM use, followed by 14.73% (2029/13,771) with multiple chronic conditions and 11.5% (2632/22,965) with no chronic conditions. The proportion of PHM use was significantly higher for individuals reporting either a single chronic condition or multiple chronic conditions than those with no chronic conditions (*P*<.001). There was no difference between the proportion of PHM use between those who reported a single chronic condition or multiple chronic conditions (*P*=.84).

**Table 1:** Demographic characteristics of respondents who reported personal health management (PHM) use by number of chronic conditions.

Variable		Without chronic condition (N=22,965)		One chronic condition (N=13,325)		Multiple chronic conditions (N=13,771)	
		All, n (%)	PHM, n (%)	All, n (%)	PHM, n (%)	All, n (%)	PHM, n (%)
**Age in years**							
	18-40	13,687 (59.60)	1644 (12.01)	4476 (33.59)	801 (17.90)	1337 (9.71)	260 (19.45)
	41-60	7233 (31.50)	816 (11.28)	5213 (39.12)	797 (15.29)	4665 (33.88)	846 (18.14)
	60+	2045 (8.90)	172 (8.41)	3636 (27.29)	398 (10.95)	7769 (56.42)	923 (11.88)
**Sex**							
	Male	10,830 (47.16)	919 (8.49)	6065 (45.52)	775 (12.78)	5733 (41.63)	902 (15.73)
	Female	12,135 (52.84)	1713 (14.12)	7260 (54.48)	1221 (16.82)	8038 (58.37)	1127 (14.02)
**Race**							
	White	17,427 (75.89)	2049 (11.76)	10,271 (77.08)	1593 (15.51)	10,541 (76.54)	1686 (15.99)
	Nonwhite	5538 (24.11)	583 (10.53)	3054 (22.92)	403 (13.20)	3230 (23.46)	343 (10.62)
**Ethnicity**							
	Not Hispanic	17,863 (77.78)	2268 (12.70)	11,442 (85.87)	1827 (15.97)	12,234 (88.84)	1904 (15.56)
	Hispanic	5102 (22.22)	364 (7.13)	1883 (14.13)	169 (8.98)	1537 (11.16)	125 (8.13)
**Born in United States**							
	Yes	17,272 (75.21)	2147 (12.43)	11,297 (84.78)	1769 (15.66)	12,155 (88.27)	1907 (15.69)
	No	5693 (24.79)	485 (8.52)	2028 (15.22)	227 (11.19)	1616 (11.73)	122 (7.55)
**Geography**							
	Midwest	4864 (21.18)	538 (11.06)	2894 (21.72)	408 (14.10)	2961 (21.50)	411 (13.88)
	Northeast	3553 (15.47)	369 (10.39)	2165 (16.25)	284 (13.12)	2249 (16.33)	316 (14.05)
	South	8032 (34.97)	829 (10.32)	4800 (36.02)	684 (14.25)	5314 (38.59)	679 (12.78)
	West	6516 (28.37)	896 (13.75)	3466 (26.01)	620 (17.89)	3247 (23.58)	623 (19.19)

**Table 2:** Socioeconomic characteristics of respondents who reported personal health management (PHM) use by number of chronic conditions.

Variable		Without chronic condition (N=22,965)		One chronic condition (N=13,325)		Multiple chronic conditions (N=13,771)	
		All, n (%)	PHM, n (%)	All, n (%)	PHM, n (%)	All, n (%)	PHM, (%)
**Education**							
	No college	8443 (36.76)	318 (3.77)	5133 (38.52)	268 (5.22)	6495 (47.16)	386 (5.94)
	College	14,522 (63.24)	2314 (15.93)	8192 (61.48)	1728 (21.09)	7276 (52.84)	1643 (22.58)
**Family income (US $)**							
	<50,000	12,023 (52.35)	844 (7.02)	7053 (52.93)	639 (9.06)	8633 (62.69)	733 (8.49)
	50,000+	10,942 (47.65)	1788 (16.34)	6272 (47.07)	1357 (21.64)	5138 (37.31)	1296 (25.22)
**Poverty**							
	Yes	4210 (18.33)	224 (5.32)	2218 (16.65)	148 (6.67)	2500 (18.15)	131 (5.24)
	No	18,755 (81.67)	2408 (12.84)	11,107 (83.35)	1848 (16.64)	11,271 (81.85)	1898 (16.84)
**Number of employees**							
	<50	15,302 (66.63)	1421 (9.29)	8467 (63.54)	1076 (12.71)	8442 (61.30)	1081 (12.81)
	51+	7663 (33.37)	1211 (15.80)	4858 (36.46)	920 (18.94)	5329 (38.70)	948 (17.79)
**Employed**							
	Employed	16,634 (72.43)	2139 (12.86)	8063 (60.51)	1448 (17.96)	4984 (36.19)	1093 (21.93)
	Unemployed	6331 (27.57)	493 (7.79)	5262 (39.49)	548 (10.41)	8787 (63.80)	936 (10.65)
**Insurance**							
	Yes	17,888 (77.89)	2462 (13.76)	11,308 (84.86)	1868 (16.52)	12,716 (92.34)	1950 (15.34)
	No	5077 (22.11)	170 (3.35)	2017 (15.14)	128 (6.35)	1055 (7.66)	79 (7.49)
**Housing**							
	Own home	12,024 (52.36)	1533 (12.75)	8231 (61.77)	1339 (16.27)	9057 (65.88)	1493 (16.48)
	Do not own	10,941 (47.64)	1099 (10.04)	5094 (38.23)	657 (12.90)	4714 (34.23)	536 (11.37)
**Food security**							
	Insecure	1291 (5.62)	84 (6.51)	991 (7.44)	69 (6.96)	1459 (10.59)	138 (9.46)
	Secure	21,674 (94.38)	2548 (11.76)	12,334 (92.56)	1927 (15.62)	12,312 (89.41)	1891 (15.36)
**Cost barriers**							
	Yes	3811 (16.59)	355 (9.32)	2672 (20.05)	342 (12.80)	2678 (19.45)	362 (13.52)
	No	19,154 (83.41)	2277 (11.89)	10,653 (79.95)	1654 (15.53)	11,093 (80.55)	1667 (15.03)
**Other barriers**							
	Yes	1825 (7.95)	356 (19.51)	1528 (11.47)	292 (19.11)	11,618 (84.37)	1666 (14.34)
	No	21,140 (92.05)	2276 (10.77)	11,797 (88.53)	1704 (14.44)	2153 (15.63)	363 (16.86)

**Table 3:** Health-related characteristics of respondents who reported personal health management (PHM) use by number of chronic conditions.

Variable		Without chronic condition (N=22,965)		One chronic condition (N=13,325)			Multiple chronic conditions (N=13,771)		
		All, n (%)	PHM, n (%)		All, n (%)	PHM, n (%)		All, n (%)	PHM, n (%)
**Health status**									
	Fair or poor	1050 (4.57)	59 (5.62)		1727 (12.96)	159 (9.21)		4472 (32.47)	458 (10.24)
	Good or excellent	21,915 (95.43)	2573 (11.74)		11,598 (87.04)	1837 (15.84)		9299 (67.53)	1571 (16.89)
**Usual place of care**									
	Yes	17,647 (76.84)	2365 (13.40)		11,736 (88.08)	1868 (15.92)		13,179 (95.70)	1980 (15.02)
	No	5318 (23.16)	267 (5.02)		1589 (11.92)	128 (8.06)		592 (4.30)	49 (8.28)
**Alcohol**									
	Non or former	7142 (31.10.)	527 (7.38)		4372 (32.81)	388 (8.87)		6192 (44.96)	579 (9.35)
	Current light	14,500 (63.14)	1928 (13.30)		8143 (61.11)	1480 (18.18)		6888 (50.02)	1326 (19.25)
	Current heavy	1323 (5.76)	177 (13.38)		810 (6.08)	128 (15.80)		691 (5.02)	124 (17.95)
**Smoking**									
	Never	15,308 (66.66)	1876 (12.26)		7500 (56.29)	1262 (16.83)		6791 (49.31)	1045 (15.39)
	Former	3478 (15.14)	465 (13.37)		3042 (22.83)	501 (16.47)		4559 (33.11)	741 (16.25)
	Current	4179 (18.20)	291 (6.96)		2783 (20.89)	233 (8.37)		2421 (17.58)	243 (10.04)
**Limits from chronic conditions**									
	Yes	1168 (5.09)	115 (9.85)		2160 (16.21)	231 (10.69)		5537 (40.21)	636 (11.49)
	No	21,797 (94.91)	2517 (11.55)		11,165 (83.79)	1765 (15.81)		8234 (59.79)	1393 (16.92)
**Depression**									
	Never	15,110 (65.80)	1510 (9.99)		7725 (57.97)	1077 (13.94)		7075 (51.38)	991 (14.01)
	Few times per year	5312 (23.13)	778 (14.65)		3497 (26.24)	608 (17.39)		3481 (25.28)	592 (17.01)
	Monthly	1202 (5.23)	185 (15.39)		843 (6.33)	152 (18.03)		927 (6.73)	153 (16.50)
	Weekly	826 (3.60)	105 (12.71)		688 (5.16)	90 (13.08)		1108 (8.05)	152 (13.72)
	Daily	515 (2.24)	54 (10.49)		572 (4.29)	69 (12.06)		1180 (8.57)	141 (11.95)
**Anxiety**									
	Never	10,306 (44.88)	809 (7.85)		5305 (39.81)	621 (11.71)		5007 (36.36)	607 (12.12)
	Few times per year	6919 (30.13)	897 (12.96)		3999 (30.01)	654 (16.35)		4038 (29.32)	668 (16.54)
	Monthly	2175 (9.47)	387 (17.79)		1297 (9.73)	264 (20.35)		1173 (8.52)	189 (16.11)
	Weekly	2104 (9.16)	356 (16.92)		1465 (10.99)	282 (19.25)		1621 (11.77)	284 (17.52)
	Daily	1461 (6.36)	183 (12.53)		1259 (9.45)	175 (13.90)		1932 (14.03)	281 (14.54)
**Moderate physical activity level**									
	Daily	3433 (14.95)	423 (12.32)		1978 (14.84)	322 (16.28)		1993 (14.47)	324 (16.26)
	Weekly	10,258 (44.67)	1557 (15.18)		5737 (43.05)	1128 (19.66)		4876 (35.41)	1006 (20.63)
	Monthly	605 (2.63)	78 (12.89)		361 (2.71)	59 (16.34)		365 (2.65)	63 (17.26)
	Annually	131 (0.57)	20 (15.27)		77 (0.58)	12 (15.58)		83 (0.60)	10 (12.05)
	Never	8538 (37.18)	554 (6.49)		5172 (38.81)	475 (9.18)		6454 (46.87)	626 (9.70)

**Figure 2:**
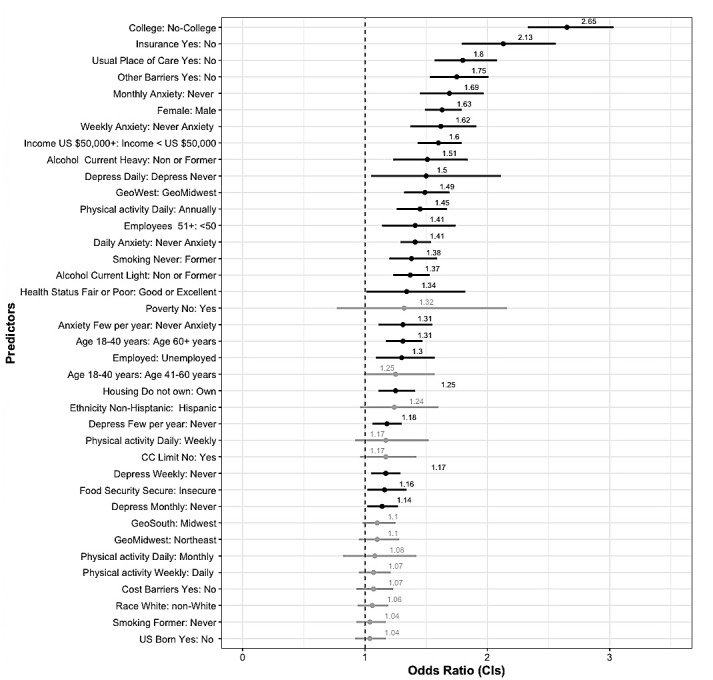
No chronic condition (CC) forest plot—multivariate logistic regression odds ratios (ORs) and 95% CIs for all predictor variables ordered by magnitude. Significant ORs are bolded. Reference categories for each predictor are on the right side of the colon.

### Personal Health Care Management Use Among Different Groups and Factors That Predict It

**Figure 3:**
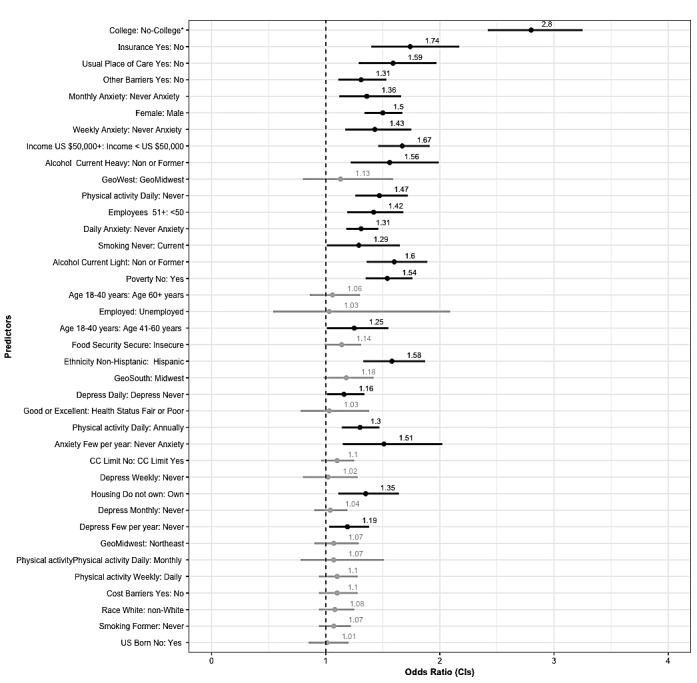
Single chronic condition (CC) forest plot—multivariate logistic regression odds ratios (ORs) and 95% CIs for all predictor variables ordered by magnitude of no chronic condition group. Significant ORs are bolded. Reference categories for each predictor are on the right side of the colon.

**Figure 4:**
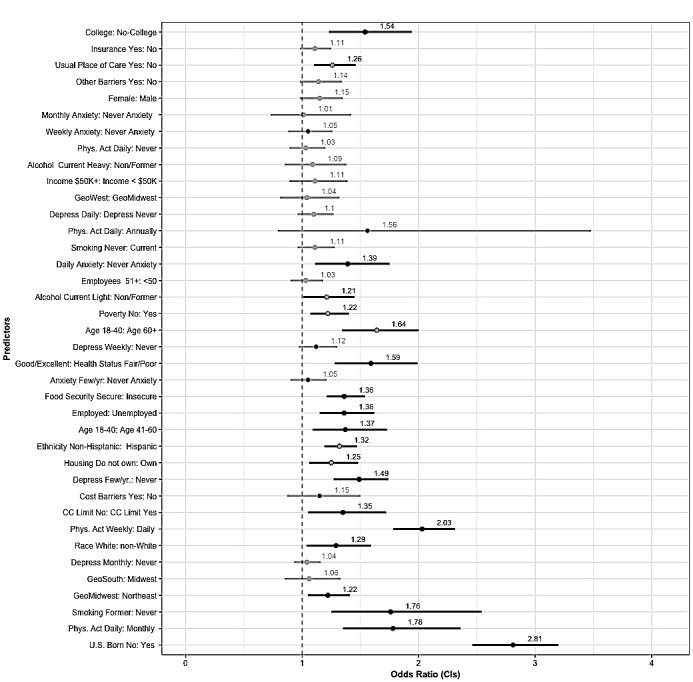
Multiple chronic conditions (CCs) forest plot—multivariate logistic regression odds ratios (ORs) and 95% CIs for all predictor variables ordered by magnitude of the no chronic condition group. Significant ORs are bolded. Reference categories for each predictor are on the right side of the colon.

Figures 2-4 show the results of the logistic regression analyses, where PHM use was the dependent variable and demographic, socioeconomic, and health-related indicators were the predictors for the groups of respondents.

### No Reported Chronic Conditions Subgroup

The lowest level of PHM use was from individuals reporting no chronic conditions. Among all factors reported in Tables 1-3, the highest proportional use of PHM was among individuals who reported monthly (17.79%, 387/2175 or weekly (16.92%, 356/2104) anxiety. Those without health insurance had the lowest proportional use of PHM (3.34%, 170/5077), followed by those without college-level education (3.76%, 318/8443) and those in poverty (5.79%, 224/4210). Educational attainment was the factor with the greatest difference in PHM use between levels. Of individuals reporting college-level education, 15.93% (2314/14522) used PHM, whereas only 3.76% (318/8443) of individuals without college-level education reported PHM use.

Figure 2 displays the odds ratios (ORs) and CIs for the variables used in predicting PHM use for respondents reporting no chronic conditions. Interpretations of the ORs using the term “likelihood” or “likely” explicitly refers to a comparison of odds used to calculate the OR for each significant variable. For this group, individuals with college-level education were over 2.6 times more likely to use PHM than those without a college-level education (OR 2.58, 95% CI 2.23-3.0). Respondents with health insurance were over 2 times more likely to use PHM than those without insurance (OR 2.11, 95% CI 1.74-2.54). Those reporting a usual place of care were nearly twice as likely to use PHM than those without a usual place of care (OR 1.81, 95% CI 1.55-2.1), and individuals reporting no other barriers to accessing health care were more likely to use PHM than those reporting such barriers (OR 1.73, 95% CI 1.49-2.02).

Women (OR 1.67, 95% CI 1.51-1.83) and individuals reporting higher family incomes (OR 1.56, 95% CI 1.37-1.77) were also more likely to use PHM. PHM use was higher for those individuals who reported having anxiety a few times per year (OR 1.28, 95% CI 1.13-1.45), having anxiety monthly (OR 1.65, 95% CI 1.39-1.96), having anxiety weekly (OR 1.58, 95% CI 1.29-1.94), and having anxiety daily (OR 1.43, 95% CI 1.12-1.84) and were more likely to use PHM than those who reported no anxiety. Individuals living in the west were more likely to use PHM than individuals in the Midwest (OR 1.52, 95% CI 1.31-1.76).

Individuals who reported engaging in moderate physical activity (PA) daily were nearly one and a half times as likely to use PHM as individuals who reported never engaging in moderate PA (OR 1.48, 95% CI 1.29-1.7). Individuals who reported working at organizations with more than 50 employees were more likely to use PHM than those working in organizations with fewer employees (OR 1.43, 95% CI 1.3-1.57), and those who reported being employed were more likely to use PHM than those who reported being unemployed (OR 1.20, 95% CI 1.03-1.38). Respondents who indicated current heavy (OR 1.56, 95% CI 1.24-1.96) or current light (OR 1.38, 95% CI 1.2-1.58) alcohol consumption were more likely to use PHM than individuals who reported not currently consuming alcohol, and individuals who reported not ever smoking were more likely to use PHM than those who reported currently smoking (OR 1.46, 95% CI 1.23-1.72).

### Single Chronic Condition Subgroup

PHM use for individuals reporting a single chronic condition was 14.97% (1996/13325). Among all factors included in the analysis (see Tables 1-3), the highest proportional use of PHM in this group was among individuals who reported incomes greater than US $50,000 per year (21.63%, 1357/6272), college-level education (21.09%, 1728/8192), and moderate PA weekly (19.66%, 1128/5737). Those without college-level education (5.22%, 268/5133), without health insurance (16.51%, 1868/11,308), and reporting being food insecure (6.96%, 69/991) had the lowest proportional use of PHM. Education level was the factor with the greatest difference in PHM use between levels. Overall, 21% of individuals reporting college-level education used PHM, whereas only 5.2% of individuals without college-level education reported PHM use.

Figure 3 illustrates the predictors of PHM use among individuals reporting a single chronic condition and is ordered by the magnitude of the no chronic condition group. Respondents with a single chronic condition who reported having a college-level education had an increased likelihood of using PHM compared with those without college-level education (OR 2.88, 95% CI 2.44-3.4). Individuals who reported having insurance (OR 1.61, 95% CI 1.26-2.06), a median family income greater than US $50 per year (OR 1.72, 95% CI 1.48-1.99), never smoking (OR 1.75, 95% CI 1.45-2.12), and having a usual place of care (OR 1.72, 95% CI 1.37-2.15) were more likely to use PHM than those who reported no health insurance, median family income of less than US $50 per year, currently smoking, and not having a usual place of health care. Adults aged 18 to 40 years were more likely to use PHM compared with adults aged 60 years and older (OR 1.59, 95% CI 1.32-1.91) and those aged 41 to 60 years (OR 1.35, 95% CI 1.18-1.54). Respondents who reported an alcohol status of current heavy (OR 1.56, 95% CI 1.32-1.84) or current light (OR 1.77, 95% CI 1.36-2.3) were more likely to use PHM than those who reported no alcohol consumption. Individuals who reported being food secure were more likely to use PHM than those who reported being food insecure (OR 1.6, 95% CI 1.16-2.0), and those respondents not in poverty were more likely to use PHM than those in poverty (OR 1.24, 95% CI 0.97-1.58). Women were more likely to use PHM compared with men (OR 1.42, 95% CI 1.25-1.62), and respondents who reported a non-Hispanic ethnicity were more likely to use PHM than those reporting a Hispanic ethnicity (OR 1.4, 95% CI 1.14-1.71). Respondents living in the western (OR 1.52, 95% CI 1.3-1.77) and southern (OR 1.27, 95% CI 1.06-1.51) regions of the United States had an increased likelihood of PHM use compared with those living in the Midwest region. Frequency of anxiety was also a predictor of PHM use.

Individuals reporting anxiety on a weekly (OR 1.46, 95% CI 1.19-1.79), monthly (OR 1.44, 95% CI 1.17-1.77), or daily (OR 1.29, 95% CI 1.01-1.65) basis were more likely to use PHM than those reporting never having anxiety. Respondents reporting daily levels of moderate PA were more likely to use PHM than those who never engage in moderate PA (OR 1.44, 95% CI 1.21-1.72), and individuals who report experiencing barriers to accessing health care were more likely to use PHM than those who reported no barriers to accessing health care (OR 1.35, 95% CI 1.14-1.59). Finally, individuals who worked at organizations with more than 50 employees (OR 1.33, 95% CI 1.19-1.49) were somewhat more likely to use PHM than those who reported working at organizations with 50 employees or less.

### Multiple Chronic Conditions Subgroup

PHM use for individuals reporting multiple chronic conditions was 14.7%. Among all factors listed in Tables 1-3, the highest proportional use of PHM in this group was among individuals who reported income greater than US $50,000 per year (25.22%, 1296/5138), had a college-level education (22.58%, 1643/7276), and had current employment (21.93%, 1093/4984). Those reporting being in poverty (5.24%, 131/2500), without college-level education (5.94%, 386/6495), without health insurance (7.48%, 79/1055), and those who were born outside the United States (7.54%, 122/1616) had the lowest proportional use of PHM. Education level and income were the 2 factors with the greatest difference in PHM use between levels. There was nearly a 17-point difference in PHM use between those who earned less than US $50 per year and those who earned more than US $50 per year and those with college-level education and those without college-level education.

Figure 4 illustrates the predictors of PHM use among individuals reporting multiple chronic conditions and is ordered by the magnitude of the no chronic condition. It indicates that college-level education was the strongest predictor of PHM use among individuals with multiple chronic conditions (OR 2.85, 95% CI 2.46-3.31), and individuals who reported family incomes greater than US $50 were more likely to use PHM than those earning less (OR 1.92, 95% CI 1.65-2.23). Similarly, individuals who reported not being in poverty were more likely to use PHM than those in poverty (OR 1.60, 95% CI 1.27-2.02). Respondents with health insurance were nearly twice as likely to use PHM than those without health insurance (OR 1.82, 95% CI 1.34-2.48), and individuals with a usual place of care had an increased likelihood of PHM use compared with those without a usual place of care (OR 1.87, 95% CI 1.28-2.72). Respondents who were born in the United States were more likely to use PHM than those who were born outside the United States (OR 1.56, 95% CI 1.23-1.97). Individuals who reported living in the west were more likely to use PHM than those in the Midwest (OR 1.47, 95% CI 1.26-1.71). Non-Hispanic respondents were more likely to use PHM than respondents who were Hispanic (OR 1.42, 95% CI 1.14-1.78), and white were more likely to use PHM than non-whites (OR 1.3, 95% CI 1.11-1.52). Individuals aged 18 to 40 years were more likely to use PHM than those older than 60 years (OR 1.54, 95% CI 1.25-1.88). Anxiety was also a predictor of PHM use. Individuals reporting daily (OR 1.45, 95% CI 1.13-1.86) or weekly anxiety (OR 1.33, 95% CI 1.06-1.66) were more likely to use PHM than individuals reporting never having anxiety. Respondents who reported never smoking were more likely to use PHM than those who reported currently smoking (OR 1.39, 95% CI 1.15-1.66). Individuals reporting current light alcohol use (OR 1.46 95% CI 1.28-1.67) or current heavy alcohol use (OR 1.42, 95% CI 1.09-1.83) were more likely to use PHM than nonalcoholic consumers. Respondents who reported working at organizations with more than 50 employees were more likely to use PHM (OR 1.26, 95% CI 1.13-1.4) than those who reported working at organizations with 50 or less employees, and individuals who reported being employed were more likely to use PHM than individuals who reported being unemployed (OR 1.27, 95% CI 1.09-1.48). Individuals who reported engaging in PA daily were more likely to use PHM than those reporting never engaging in PA (OR 1.21, 95% CI 1.0-1.47).

### Comparisons Across Groups

For the logistic regressions, college education consistently had the largest ORs, with analyses from all subgroups reporting respondents with a college education being nearly 3 times as likely to report PHM use. Interestingly, women reporting no chronic conditions or 1 chronic condition were more likely to use PHM than men, and only among respondents reporting multiple chronic conditions was race found to be a significant predictor. Table 4 reports the ORs and CIs for all significant factors across the no chronic condition, single chronic condition, and multiple chronic conditions groups. There were few differences in significant predictors between the 3 models. On the basis of nonoverlapping CIs, the odds of using PHM were higher for those with no chronic conditions who reported no other barriers to accessing care than those reporting the same with 1 or more chronic conditions. Similarly, based on a slight overlap in CIs (0.05), the odds of using PHM among those with higher family incomes were higher for those with multiple chronic conditions compared with those with no chronic conditions.

**Table 4:** Comparison of patient health management (PHM) use by significant characteristics between respondents reporting no chronic conditions, 1 chronic condition, and multiple chronic conditions. Italics indicate nonsignificant findings.

Variable category and name			No chronic condition (n=22,929), OR^a^ (95% CI)	One chronic condition (n=12,415), OR (95% CI)	Multiple chronic conditions (n=12,470), OR (95% CI)
**Demographics**					
	**Age in years**				
		18-40	1.3 0 (1.09 -1.57)^b^	1.58 (1.33 -1.87)^b^	1.64 (1.34-2.00)^b^
	**Sex**				
		Female	1.63 (1.49-1.79)	1.5 0 (1.34-1.67)	*1.04 (0.93-1.16*)
	**Race**				
		White	*1.06 (0.94-1.19)*	*1.08 (0.94-1.25)*	1.26 (1.11-1.46)
	**Ethnicity**				
		Not Hispanic	1.16 (1.02-1.34)	1.35 (1.11-1.64)	1.39 (1.11-1.75)
	**US born**				
		Yes	*1.04 (0.92-1.17)*	*1.01 (0.85-1.20)*	1.54 (1.23 -1.94)
	**Geography**				
		South	*1.11 (0.98-1.25)*	1.19 (1.03-1.38)	*1.03 (0.89-1.20)*
		West	1.49 (1.32-1.69)	1.47 (1.26-1.72)	1.49 (1.27 -1.74)
**Socioeconomic status**					
	**Education**				
		College	2.65 (2.33-3.03)	2.80 (2.42-3.25)	2.81 (2.46 -3.20)
	**Family income**				
		US $50,000+	1.60 (1.43-1.79)	1.67 (1.46 -1.91)	2.03 (1.78 -2.31)
	**Poverty**				
		No	1.31 (1.11-1.55)	1.25 (1.01 -1.55)	1.59 (1.28 -1.99)
	**Number of employees**				
		51+	1.41 (1.29-1.54)	1.31 (1.18 -1.46)	1.32 (1.19-1.47)
	**Employed**				
		Yes	1.25 (1.11 -1.41)	1.16 (1.01 -1.34)	1.22 (1.07-1.40)
	**Insurance**				
		Yes	2.13 (1.79 -2.56)	1.74 (1.40-2.17)	1.78 (1.35 -2.36)
	**Housing**				
		Do not own	1.17 (1.05-1.29)	*1.10 (0.96-1.25)*	*1.03 (0.90-1.18)*
	**Food security**				
		Secure	1.51 (1.15-2.02)	1.51 (1.15-2.02)	*1.11 (0.89-1.39)*
	**Other barriers**				
		Yes	1.75 (1.53 -2.01)	1.31 (1.11-1.53)	1.22 (1.05 -1.41)
**Health-related characteristics**					
	**Health status**				
		Fair or poor	1.34 (1.01 -1.82)	*1.06 (0.86-1.30)*	*1.05 (0.90-1.21)*
	**Usual place** **of** **care**				
		Yes	1.82 (1.56-2.08)	1.59 (1.29 -1.97)	1.76 (1.25-2.54)
	**Alcohol**				
		Current light	1.37 (1.23-1.53)	1.54 (1.35-1.76)	1.36 (1.21-1.54)
		Current heavy	1.51 (1.23-1.84)	1.56 (1.22-1.99)	1.35 (1.05 -1.72)
	**Smoking**				
		Never	1.38 (1.20-1.59)	1.6 0 (1.36-1.89)	1.36 (1.15 -1.62)
	**Depression**				
		Few times per year	1.14 (1.02-1.27)	*1.04 (0.90-1.19)*	*1.11 (0.96-1.28)*
		Daily	1.5 0 (1.05 -2.11)	*1.13 (0.80-1.59)*	*1.15 (0.87-1.50)*
	**Anxiety**				
		Few times per year	1.31 (1.17-1.47)	*1.14 (0.99-1.31)*	*1.12 (0.97-1.30)*
		Monthly	1.69 (1.45 -1.97)	1.36 (1.12 -1.66)	*1.06 (0.85-1.33)*
		Weekly	1.62 (1.37 -1.91)	1.43 (1.17 -1.75)	1.29 (1.04 -1.59)
		Daily	1.41 (1.14 -1.74)	1.29 (1.01 -1.65)	1.37 (1.09-1.73)
	**Moderate physical activity level**				
		Daily	1.45 (1.26-1.67)	1.42 (1.19-1.68)	1.25 (1.06-1.48)

^a^OR: odds ratio.

^b^The age range of 18-40 years had significantly higher odds than both other age categories.

## Discussion

### Principal Findings

Web-based interactions between patients and health organizations related to the access of health information are becoming a focused area of attention. Between 2009 and 2014, there was an increase in the use of secure email to communicate with HCPs [38]. The impact of patient access and use of health information has been thoroughly documented in the literature [39]. However, more detailed studies have shown that individuals who engaged with PHM were more likely to report an improved experience of care, improved outcomes, and improved health literacy. Despite increased attention to engaging patients and families in health decision making by providing access to electronic health information, there has been an overall low level of use of these tools by patients for PHM. Findings from this study support that assertion in that it found only 12.2% of American adults reported PHM use between 2009 and 2014, and the percentage of PHM use among this group has remained relatively stable over this period—only increasing slightly from less than 2% points from 2009 to 2014. This slight increase may be partly explained by the adoption of EHR systems that have the ability to engage patients. For example, the rate of adoption of systems with patient engagement functionality increased from 28% to 40% between 2009 and 2012 [40].

This study demonstrated that there are differences in PHM use across demographic, socioeconomic, and health-related individual-specific factors, and the proportions of persons reporting PHM use are different across groups with no chronic conditions versus groups with 1 or multiple chronic conditions. A greater proportion of individuals with chronic health conditions reported PHM use compared with those without chronic health conditions, and this finding is consistent with prior research [41]. The use of PHM by a greater proportion of individuals suffering from chronic conditions may be partly explained by clinical need. Individuals with chronic conditions may have complex treatment plans, they seek health care more frequently, and the management of chronic conditions typically requires medication management [42]. Thus, individuals accessing and managing care more frequently may result in greater use of scheduling appointments on the Web, requesting prescription refills on the Web, or communicating with HCPs on the Web.

### Demographic Factors

Demographic factors clearly influenced PHM use across all 3 groups. Individuals who were younger, non-Hispanic, and who lived in the west reported the greatest levels of PHM use. Individuals who reported being born in the United States with multiple chronic conditions were more likely to use PHM compared with the other 2 models. Previous research has shown that white [7,25,28,29] and non-Hispanic individuals are more likely to use PHM than other racial or ethnic groups [23,25]. Our findings suggest a relationship between complexity of condition and PHM use. Previous research has also demonstrated that patients who are younger and non-Hispanic are more likely to be engaged in their health care. Our results confirm this previous research as related to individuals who reported a single chronic condition. Among the no chronic condition and single chronic condition groups, women were more likely to use PHM than men, and this finding is also consistent with previous research [7,23,25].

### Socioeconomic Factors

Socioeconomic factors were the most predictive of PHM use across all 3 groups. Across all the groups, those with a college-level education were more than 2.65 times as likely to use PHM. Research has shown that there is a relationship between education level and health literacy and that health literacy increases a patient’s engagement in health care decision making [28]. An individual’s level of education is also associated with socioeconomic status. As socioeconomic status impacts an individual’s health literacy, access to routine health care services, and also access to internet and computer technology, education level is a critical determinant in whether an individual emails an HCP, schedules an appointment on the Web, or requests a prescription refill on the Web [13,25]. There are clear disparities in PHM use based on education level. This issue deserves focused attention to ensure the disparities do not continue to widen over time. Similarly, insurance coverage and family income were among the strongest predictors of PHM use across the 3 groups, and these factors have been previously shown to be associated with patient engagement [7,43,44]. These findings are consistent with prior research regarding disparities in the use of patient-centered technology to connect patients to electronic health information [3,28]. Having a lower socioeconomic status may indicate the lack of internet access that would enable an individual to connect to their electronic health information [45]. Lower socioeconomic status may also indicate lack of employment and health insurance, thus an increased likelihood of encountering barriers to accessing health care and therefore electronic health information provided by health care organizations [13,25].

### Health-Related Factors

Another predictor of PHM is having a usual place for receiving health care. Research has shown there is variation in EHR adoption nationally [46,47]. Different rates of EHR system adoption among both inpatient and ambulatory health care organizations may be impacting the PHM use. As PHM is associated with communicating with HCPs and using technology associated with health care organizations, having a usual source for receiving health care and therefore potentially having stronger relationships with HCPs on the surface would facilitate increased use of PHM [48]. Having a usual place of care also may indicate that these individuals routinely attend the same place for their clinical encounters and potentially obtain care from the same clinicians. This routine care from the same clinic and care team indicates that having an organization that one may consider a health care *home* produces an environment that promotes communication between clinicians and patients through technology.

Engaging in moderate PA was also associated with increased PHM use. Research has shown that there is a relationship between health status and patient engagement, and these findings suggest that individuals who are more active and therefore potentially healthier are more likely to engage in managing their health information through technology.

Interestingly, individuals with 1 chronic condition who reported current light alcohol use and daily moderate PA were more likely to use PHM than those who reported never consuming alcohol and never engaging in moderate PA. These findings can partly be explained by the association between health status and PHM use. Individuals who reported healthier lifestyle behavior such as PA, moderate alcohol consumption, and not smoking were also more likely to use PHM. Research has shown that patients who are less physically active are more likely to be disengaged in their health care [49]. Individuals who report never participating in moderate PA are less likely to use PHM than individuals who participate in moderate PA daily, which indicates a relationship between a general health-related lifestyle or physical ability and use of technology for accessing personal health information. Research has shown that there is a relationship between social determinants of health such as access to resources and an individual’s level of PA [50].

Individuals who reported daily and weekly anxiety were more likely to use PHM than those who reported never having anxiety across all 3 subgroups. Previous research has found a relationship between anxiety and use of technology [51-53]. Research related to the Unified Theory of Acceptance and Use of Technology found that increased anxiety levels related to use of computers is negatively associated with behavioral intention to use technology [53]. Our findings suggest that there is an association between individual anxiety level and use of technology to email HCPs, schedule appointments on the Web, or request prescription refills on the Web. This may be partly explained by the relationship between chronic disease, stress, and anxiety [54]. Research has shown that *health anxiety* influences patient care and information-seeking behavior [55]. This finding suggests an association between individual concerns and individual behaviors surrounding electronic access and use of heath information. More anxious and more depressed patients are significantly more likely to use PHM. As anxiety and depression are often related, it makes some sense that these groups are seeking some reassurance from using PHM in any of its forms. It is important to reiterate that PHM consists of texting providers, refilling prescriptions, and scheduling appointments. It is possible that these individuals are attempting to consult with a provider to address their anxiety and depressive state.

### Observations of Significant Factors Among Groups

This study demonstrated a small number of differences between the models for respondent subgroups. The odds of using PHM were higher among those without a chronic condition and those who do not experience barriers to accessing health care. This finding suggests that those individuals who do not experience challenges to accessing care and have chronic conditions may be using PHM to complement their typical access to HCPs or assist in managing their disease. Certain demographic factors also differed across models. Women without chronic conditions were more likely to use PHM than women with either 1 or multiple chronic conditions, and this finding is consistent with previous research [7,23,25,56].

### Limitations

There are several limitations of this study. The findings are limited by the use of existing data collected through the NHIS survey for a purpose different from this study. The study depended upon the use of the standardized items included through self-reporting in the data collection process. Self-reported survey data have the potential to be biased by social desirability, leading to providing answers that the respondent perceives as more desirable. This could have led to an overestimate of the PHM use as compared with measurement of actual use. This study also relied on combining multiple years of NHIS data and adjusting sample weights to account for pooling data across years. This also could have led to an overestimation of PHM use based on certain factors. Our findings point to a direction for further, more sophisticated analysis of these data to further explore the findings. Another potential limitation of this study is the combined measure of PHM. The measure was constructed to reflect personal use of technology related to health care organizations, but the construction of the measure was limited to the 3 predefined items related to technology use. Furthermore, the questions asked only if the respondent had ever engaged in the activity, so responses did not reflect the intensity of use. This may lead to the underestimation of actual use, as respondents who reported engaging in the behavior based on only 1 incident are lumped with those who displayed the same behavior frequently. We conclude that the questions related to emailing HCPs, requesting prescription refills on the Web, and scheduling appointments are aggregable into a single PHM measure because of their high rates of co-occurrence in the dataset. There may also be additional measures that also reflect PHM that were not included in the dataset such as patient review of laboratory tests or visit summaries that were not included in the survey and have been reported previously. Leaving out these frequently reported behaviors could lead to an underestimate of use. These limitations of the dataset may separately lead to either over or underestimates of PHM use. We argue that this implies that our reported results are reasonable estimates but may have wider CIs than calculated. The NHIS did not collect information related to PHM in 2010, which may impact the overall proportional use of PHM across all years, but this was addressed by excluding this year from the analytic dataset.

### Conclusions

The purpose of this research was to describe the overall utilization of PHM and compare individual characteristics associated with PHM in groups with no reported chronic conditions, with 1 chronic condition, and with 2 or more such conditions. The results indicate that the overall usage of PHM is not increasing along with the increased use of EHRs in the United States, even when clinical providers and hospitals are offering PHM features to patients. The overall use of PHM has increased slightly since 2009, but individuals reporting 1 or more chronic conditions used PHM at higher rates than individuals reporting no chronic conditions. The findings of this study also illustrated the disparities in PHM use across multiple factors, including economics and education in a nationally representative sample of individuals. These findings provide further evidence of the challenge associated with engaging patients through the use of electronic health information as the health care industry continues to evolve. Although health care organizations continue to adopt electronic modes of communication to facilitate interactions between patients and health care organizations, there are significant gaps related to the use of these tools for connecting consumers to health information. For each chronic condition category analyzed, demographic and socioeconomic factors appear to be driving PHM use. Research has demonstrated that patient-centered technologies are associated with improved clinical outcomes, patient experience, and health literacy. If action is not taken to address disparities in PHM use, individuals with lower socioeconomic status are at risk of seeing gaps in health disparities widen. In the short term, it is imperative that health care organizations develop initiatives aimed at promoting adoption of these tools by all individuals, regardless of socioeconomic status. Initiatives must be sensitive to health literacy, race, ethnicity, and other social determinants of health in their design if substantial progress in PHM use is to be achieved. In the long term, technologies that support PHM use must be designed to better meet the needs of patient populations. The current rate of use reflects the general lack of adoption of these tools, which can be partly explained by the lack of interest or need to access electronic health information on the Web. If PHM use is to increase over time, there needs to be better access to health care information across the continuum of care and more integration of tools and information related to personal fitness, diet, and lifestyle into the systems that support PHM use. Attention needs to be placed on developing technology solutions that meet the needs of all individuals regardless of educational achievement.

Future studies should investigate the relationship between PHM use and clinical outcomes across different chronic conditions, as well as the relationship between PHM use and usability of systems that support PHM-related functions. An additional level of analysis is warranted in which multiple significant predictors are clustered, rather than having 22 to 25 variables analyzed separately.

Previous research has indicated a relationship between the PHM and improved patient satisfaction, care outcomes, and knowledge. This work demonstrated that even though there was an overall low level of PHM use, there were clear disparities across demographic, socioeconomic, and health-related variables. The research showed that having a chronic condition is not the characteristic that best explains PHM use.

## Abbreviations

CMS: Centers for Medicare and Medicaid Services
EHR: electronic health record
HCP: health care provider
IHIS: Integrated Health Interview Series
NHIS: National Health Interview Survey
OR: odds ratio
PA: physical activity
PHM: personal health management
